# Ethanol’s interaction with BK channel α subunit residue K361 does not mediate behavioral responses to alcohol in mice

**DOI:** 10.1038/s41380-023-02346-y

**Published:** 2023-12-22

**Authors:** Agbonlahor Okhuarobo, Max Kreifeldt, Pauravi J. Gandhi, Catherine Lopez, Briana Martinez, Kiera Fleck, Michal Bajo, Pushpita Bhattacharyya, Alex M. Dopico, Marisa Roberto, Amanda J. Roberts, Gregg E. Homanics, Candice Contet

**Affiliations:** 1 The Scripps Research Institute, Department of Molecular Medicine, La Jolla, CA USA; 2 University of Tennessee Health Science Center, Department of Pharmacology, Addiction Science, and Toxicology, Memphis, TN USA; 3 The Scripps Research Institute, Animals Models Core Facility, La Jolla, CA USA; 4 University of Pittsburgh, Department of Anesthesiology and Perioperative Medicine, Pittsburgh, PA USA

**Keywords:** Neuroscience, Genetics

## Abstract

Large conductance potassium (BK) channels are among the most sensitive molecular targets of ethanol and genetic variations in the channel-forming α subunit have been nominally associated with alcohol use disorders. However, whether the action of ethanol at BK α influences the motivation to drink alcohol remains to be determined. To address this question, we first tested the effect of systemically administered BK channel modulators on voluntary alcohol consumption in C57BL/6J males. Penitrem A (blocker) exerted dose-dependent effects on moderate alcohol intake, while paxilline (blocker) and BMS-204352 (opener) were ineffective. Because pharmacological manipulations are inherently limited by non-specific effects, we then sought to investigate the behavioral relevance of ethanol’s direct interaction with BK α by introducing in the mouse genome a point mutation known to render BK channels insensitive to ethanol while preserving their physiological function. The BK α K361N substitution prevented ethanol from reducing spike threshold in medial habenula neurons. However, it did not alter acute responses to ethanol in vivo, including ataxia, sedation, hypothermia, analgesia, and conditioned place preference. Furthermore, the mutation did not have reproducible effects on alcohol consumption in limited, continuous, or intermittent access home cage two-bottle choice paradigms conducted in both males and females. Notably, in contrast to previous observations made in mice missing BK channel auxiliary β subunits, the BK α K361N substitution had no significant impact on ethanol intake escalation induced by chronic intermittent alcohol vapor inhalation. It also did not affect the metabolic and locomotor consequences of chronic alcohol exposure. Altogether, these data suggest that the direct interaction of ethanol with BK α does not mediate the alcohol-related phenotypes examined here in mice.

## Introduction

Calcium- and voltage-activated, large conductance potassium (BK) channels are one of the primary molecular targets of ethanol in the brain [1–3]. Depending on multiple molecular determinants (e.g., intracellular calcium concentration, alternative splicing, subunit composition, posttranslational modifications, lipid microenvironment), ethanol can either potentiate or reduce BK channel-mediated currents (see [4] for review). Polymorphisms in *KCNMA1*, the gene encoding the channel-forming α subunits, were associated with alcohol use disorders (AUD) at nominal significance in several human genomic studies (see [5] for review). However, whether the action of ethanol on mammalian BK channels mediates the behavioral effects of ethanol and influences the motivation to drink alcohol remains to be determined. Filling this gap of knowledge has critical implications for the understanding and treatment of AUD.

Until now, the contribution of BK channels to the behavioral effects of ethanol has been studied by genetically manipulating the channel-forming α subunit in worms and flies, and the auxiliary β subunits in mice. Studies in invertebrates showed that BK α mediates the intoxicating effects of ethanol in worms [6, 7] and rapid tolerance to ethanol-induced sedation and increased seizure susceptibility in flies [8–11]. In mice, deletion of BK β4 promotes rapid tolerance to the locomotor depressant effect of ethanol [12] and attenuates ethanol drinking escalation in mice exposed to chronic intermittent ethanol (CIE) vapor inhalation [13]. Conversely, deletion of BK β1 accelerates drinking escalation in CIE-exposed mice [13] and reduces chronic tolerance to ethanol-induced sedation and hypothermia [14]. These findings suggest that BK auxiliary subunits play a role in the adaptive response to chronic ethanol exposure in mammals but fail to provide a direct insight into the role of ethanol’s interaction with BK α subunit in alcohol-related behaviors.

In the present study, we sought to establish whether the action of ethanol at BK α contributes to alcohol drinking and other responses elicited by alcohol in mice. We first used pharmacological modulators of BK channels and then turned to a more specific genetic approach to block the interaction of ethanol with BK α without affecting basal BK channel function. Using recombinant BK channels expressed in *Xenopus* oocytes, residue K361 in BK α cytoplasmic tail domain was shown to play a key role in ethanol recognition as hydrogen bond donor and for the ensuing increase in the channel’s open probability [15]. Importantly, while the K361N substitution confers refractoriness to 100 mM ethanol, it does not alter basal steady-state activity of BK channels, nor their sensitivity to the BK channel primary endogenous activators: voltage and intracellular calcium [15]. It therefore represents a powerful tool to probe the in vivo relevance of ethanol’s direct interaction with BK α using gene editing in mice. Our results demonstrate that preventing ethanol from interacting with BK α K361 does not alter several behavioral effects of acute or chronic alcohol exposure, including the motivation to drink alcohol.

## Materials and methods

### Animals

C57BL/6J mice were obtained from The Jackson Laboratory or from The Scripps Research Institute (TSRI) rodent breeding colony. BK α K361N knockin (KI) mice were generated at the University of Pittsburgh. Breeders were sent to TSRI, where a colony was maintained by mating heterozygous (Het) males and females such that experimental mice were littermates. KI mice were backcrossed to C57BL/6J mice every 1-2 years to prevent genetic drift.

Mice were maintained on a 12 h/12 h light/dark cycle. Food (Teklad LM-485, Envigo) and acidified or reverse osmosis purified water were available *ad libitum*. Sani-Chips (Envigo) were used for bedding substrate. Behavioral testing was started when mice were at least 10 weeks old. Mice were individually housed for drinking experiments and metabolic and activity tracking, but group-housed otherwise. Testing was conducted during the dark phase of the circadian cycle, except for conditioned place preference, which was conducted during the light phase.

All procedures adhered to the National Institutes of Health Guide for the Care and Use of Laboratory Animals and were approved by the Institutional Animal Care and Use Committees of the University of Pittsburgh and TSRI.

### Experimental cohorts

Independent cohorts of C57BL/6J males were used to test the effects of penitrem A on tremors (*n* = 28, 6-8 mice per dose), alcohol drinking (*n* = 20) and saccharin drinking (*n* = 20), as well as the effects of paxilline (*n* = 18) and BMS-204352 (*n* = 30) on alcohol drinking. Investigators were blind to treatment during data collection.

To evaluate the effect of BK α K361N substitution, body weights were collected in 6-week-old males (WT, *n* = 7; Het, *n* = 12; KI, *n* = 6). Brains from experimentally naïve adult males and females (*n* = 3 per sex and per genotype) were processed for BK α immunoblotting. Electrophysiological recordings were obtained from experimentally naïve adult males (WT paxilline, *n* = 6 cells from 3 mice; WT ethanol, *n* = 12 cells from 4 mice; KI ethanol, *n* = 10 cells from 4 mice). Separate cohorts of males were tested for ethanol clearance rate (WT, *n* = 3; Het, *n* = 4; KI, *n* = 4), ethanol-induced ataxia, sedation, and hypothermia (WT, *n* = 10; Het, *n* = 11; KI, *n* = 8), ethanol-induced analgesia (WT, *n* = 7; Het, *n* = 9; KI, *n* = 7), and conditioned place preference (WT, *n* = 11; KI, *n* = 15). Three independent cohorts of mice, each containing an equivalent number of WT and KI males, were tested for limited-access alcohol drinking acquisition and CIE-induced escalation, and their data were pooled for analysis (WT, *n* = 19; KI, *n* = 15). This experiment was repeated in another set of three independent cohorts of mice, each containing an equivalent number of WT and KI males and females, and the data were pooled by sex for analysis (WT males, *n* = 21; KI males, *n* = 20; WT females, *n* = 21; KI females, *n* = 22). Two additional cohorts of males were used to measure metabolism/activity (WT, *n* = 8; KI, *n* = 7) and circadian rhythmicity (WT, *n* = 8; KI, *n* = 8) during withdrawal from CIE. Continuous- and intermittent-access alcohol drinking were first tested in a cohort containing both males and females (WT males, *n* = 5; KI males, *n* = 7; WT females, *n* = 6; KI females, *n* = 6), and later repeated in a separate cohort of females (WT females, *n* = 10; KI females, *n* = 9). Investigators were blind to genotype during data collection.

### Drugs

Penitrem A was purchased from Sigma (P3053) for tremor assessment and from Enzo Life Sciences (BML-KC157) for drinking experiments. It was dissolved in dimethylsulfoxide (DMSO) at 10 mg/mL and diluted in saline for intraperitoneal (i.p.) injection (0.1 mL per 10 g body weight). The final concentration of DMSO was 50% for tremor assessment and 10% for drinking experiments.

Paxilline was purchased from Sigma (P2928), dissolved in DMSO at 10 mM and diluted in phosphate-buffered saline (137 mM NaCl, 2.7 mM KCl, 1.8 mM KH_2_PO_4_, 10.1 mM Na_2_HPO_4_, pH 7.4) for i.p. injection (1:2000 for 22 µg/kg dose, 1:400 for 110 µg/kg dose, 1:80 for 550 µg/kg dose). Each dose was tested on a different week. Doses were tested in ascending order, and vehicle and drug treatments were counterbalanced over two consecutive days for each dose. This dose range was selected based on pilot testing that indicated reduced mobility at 1.1 mg/kg and tremors at 4.4 mg/kg, which would have confounded drinking behavior, as well as on reported anticonvulsant properties of ultra-low-dose paxilline [16].

BMS-204352 was purchased from Sigma (SML1313), dissolved in DMSO at 16 mg/mL and diluted in Tween-80:saline at a 1:1:80 ratio for i.p. injection. A dose of 2 mg/kg was selected based on its ability to reverse behavioral abnormalities in *Fmr1* mutant mice [17, 18]. This dose did not produce a noticeable effect on mouse behavior (e.g., hyperactivity or sedation).

Ethyl alcohol 200 proof (PHARMCO-AAPER, 111000200) was used for i.p. administration (diluted in saline) and drinking solutions (diluted in acidified or reverse osmosis purified water). Ethyl alcohol 95% (PHARMCO-AAPER, 111000190) was used for vapor inhalation.

### Generation of BK α K361N KI mice

KI mice were produced using CRISPR/Cas9 technology as previously described in detail [19]. Briefly, a sgRNA targeting *Kcnma1* in exon 9 near the intended mutation site was identified using the CRISPR Design Tool [20]. Two overlapping PCR primers (forward: GAAATTAATACGACTCACTATAGGAGTGTCTCTAACTTCCTGAGTTTTAGAGCTAGAAATAGC; R: AAAAGCACCGACTCGGTGCCACTTTTTCAAGTTGATAACGGACTAGCCTTATTTTAACTTGCTATTTCTAGCTCTAAAAC) were used to generate a T7 promoter containing sgRNA template as described [21]. The sgRNA and Cas9 mRNA were produced by in vitro translation, purified (MEGAclear Kit, Ambion), ethanol precipitated, and resuspended in DEPC-treated water. A 120-nucleotide single-stranded DNA repair template oligonucleotide harboring the desired mutations in exon 9 of *Kcnma1* was purchased as Ultramer DNA (Integrated DNA Technologies, Coralville, IA). sgRNA (25 ng/µl), Cas9 mRNA (50 ng/µl), and repair oligo (100 ng/µl) were combined and injected into the cytoplasm of C57BL/6 J one-cell embryos as described [22]. Embryos that survived injection were transferred to the oviduct of day 0.5 post-coitum pseudo-pregnant CD-1 recipient females. Pups resulting from injected embryos were screened for DNA sequence changes in exon 9 of the *Kcnma1* gene by PCR/DNA sequence analysis. A male founder mouse harboring the desired changes was mated to C57BL/6J females to establish the KI line. The *Kcnma1* exon 9 containing amplicon from all Het F1 animals that were shipped for the TSRI breeding colony were sequenced to verify the fidelity of the mutated locus. The founder mouse harbored no off-target mutations (data not shown) in any of the top 7 off-target sites predicted by the Off –Targets tool of the Cas9 Online Designer [23].

Mice were genotyped by subjecting tail clip lysates to polymerase chain reaction (PCR) using a pair of primers (forward: GCTTTGCCTCATGACCCTCT; reverse: TGAACAAGGGTGCTGCTTCA) that amplifies a 450-bp fragment of the *Kcnma1* gene. The PCR products were then digested with Tru1I and the resulting fragments were visualized by electrophoresis in an ethidium bromide-stained agarose gel. Tru1I digestion yields two fragments (107 + 343 bp) in the wild-type (WT) allele and three fragments (107 + 149 + 194 bp) in the KI allele (see KI-specific Tru1I site in Fig. 2A).

To verify that the mutation was also present in *Kcnma1* mRNA, RNA was isolated from a KI mouse brain hemisphere using the RNeasy Plus Universal Mini Kit (Qiagen, 73404), 2 µg of RNA was reverse-transcribed using the Transcriptor First Strand cDNA Synthesis Kit with random hexamer primers (Roche, 04379012001), and a 370-bp fragment (nucleotides 1304-1673 of NM_010610.3) was amplified from the resulting cDNA. This fragment was cloned into pBluescript II and sequenced with a T3 primer (Genewiz).

### Immunoblotting

Mice were euthanized by cervical dislocation. Brains were flash frozen in isopentane (Sigma-Aldrich), then stored at −80 °C. The right hemisphere of each brain was homogenized in 7 mL of ice-cold 0.25 M sucrose solution using a Tissue-Tearor (Biospec Products Model 985370-395). The homogenate was centrifuged at 1350 x g for 10 min at 4 °C and the supernatant was collected. The pellet was homogenized in 4 mL 0.25 M sucrose and centrifuged at 1350 x g for 10 min at 4 °C. Supernatants were combined in 50 mM Tris-HCl, pH 7.5 and 1 mM EDTA to a total volume of 12 mL. A 1-mL aliquot was centrifuged at 40,000 x *g* for 30 min at 4 °C and the resulting pellet was resuspended in 250 μL 0.32 M sucrose using an electric pestle (VWR 47747-370). Protein concentrations were determined using the Pierce BCA Protein Assay Kit (ThermoFisher Scientific). Samples were stored at −80 °C until diluted in 4x Laemmli Sample Buffer containing 10% 2-mercaptoethanol (Bio-Rad Laboratories). The diluted samples were heated at 37 °C for 45 min, then cooled back to room temperature. Next, a volume corresponding to 10 μg of protein was loaded into a 4–20% Mini-PROTEAN TGX Stain-Free Gel (Bio-Rad Laboratories) and electrophoresed at 80 V for 5 min then 180 V through the gel using a Mini-PROTEAN Tetra Cell electrophoresis apparatus (Bio-Rad Laboratories). The gel was then activated for 1 min using a ChemiDoc MP System (Bio-Rad Laboratories). The proteins were transferred to a PVDF Immun-Blot membrane (Bio-Rad Laboratories) using a Mini Trans-Blot cell (Bio-Rad Laboratories) running at 100 V for 1 h in a cold room. After transfer, the blot was imaged using the stain-free setting in a ChemiDoc MP System to quantify the amount of protein present in each lane. The membrane was blocked for 1 h at room temperature in TBST (20 mM Tris pH 7.5, 500 mM NaCl, and 0.1% Tween 20) with 5% bovine serum albumin (BSA) fraction V (ThermoFisher Scientific). Next, the membrane was incubated overnight at 4 °C with the primary antibody (Anti-Slo1 clone L6/60, Millipore Sigma MABN70, 1:1000 [24]) diluted in TBST with 2.5% BSA. Next, the membrane was washed at room temperature 3 × 10 min with TBST, incubated for 1 h at room temperature in TBST containing a 1:5000 dilution of goat anti-mouse IgG (H + L) horseradish peroxidase-conjugated antibody (Bio-Rad Laboratories #170-6516), then washed at room temperature 3 × 10 min with TBST. Finally, the membrane was bathed with 2 mL of SuperSignal West Pico PLUS Chemiluminescent Substrate (ThermoFisher Scientific) and imaged using a ChemiDoc MP System. The density of immunolabeling, as well as total protein in corresponding lanes, was acquired using Image Lab Software (version 6.1; Bio-Rad Laboratories) with background subtraction enabled, using a disc size of 10 mm for immunolabeling and 70 mm for total protein. The immunoblotting signal in each lane was normalized to total protein density to adjust for variability in protein loading and transfer. All samples were run on the same gel/blot to minimize variability in transfer and immunodetection.

### In situ hybridization

Custom locked nucleic acid (LNA) oligoprobes labeled with digoxigenin in 3’ and 5’ were obtained from Exiqon (Denmark). The sequences were as follows:

STREX (batch 620646) /5DigN/ATGCTCGTCTCATTCTCTTGTA/3Dig_N/

ALCOREX (batch 620645) /5DigN/TTCCTGATTGACTGATACCA/3Dig_N/

The same protocol and reagents as in [25] were used to prepare brain sections and label *Kcnma1* splice variants. The probes were hybridized overnight at 48°C at a final concentration of 5 nM.

### Electrophysiological recordings

Recordings were obtained from sagittal brain slices (300-μm thick) containing the medial habenula (mHb). The slices were cut in oxygenated (95% O_2_/5% CO_2_), ice-cold high-sucrose solution (pH 7.3-7.4, 206.0 mM sucrose, 2.5 mM KCl, 0.5 mM CaCl_2_, 7.0 mM MgCl_2_, 1.2 mM NaH_2_PO_4_, 26 mM NaHCO_3_, 5 mM glucose, 5 mM HEPES) using a vibratome (Leica VT1200S) and then incubated in an oxygenated recovery solution (130 mM NaCl, 3.5 mM KCl, 2 mM CaCl_2_, 1.25 mM NaH_2_PO_4_, 1.5 mM MgSO_4_, 24 mM NaHCO_3_, 10 mM glucose, and 1 mM kynurenic acid) at 32˚C for 30 min. After recovery, the slices were kept in oxygenated artificial cerebrospinal fluid (aCSF: 130 mM NaCl, 3.5 mM KCl, 2 mM CaCl_2_, 1.25 mM NaH_2_PO_4_, 1.5 mM MgSO_4_, 24 mM NaHCO_3_, and 10 mM glucose) at room temperature for at least 30 min until recording started [26, 27].

mHb neurons were visualized using an upright microscope (Olympus BX51WI) with infrared-differential interference contrast (IR-DIC) optics, a w40 or w60 water immersion objective, and a CCD camera (EXi Aqua, QImaging). Whole-cell recordings of the current-voltage relationships (IVs) were performed in the current clamp mode at a sampling rate per signal of 50 kHz and low-pass filtered at 10 kHz, using a Multiclamp 700B amplifier, Digidata 1440 A and pClamp 10.2 software (all Molecular Devices). The neurons were held at –60 mV, and the recording glass electrodes (3-6 MΩ resistance) were filled with K-gluconate internal solution: 145 mM K-gluconate, 5 mM EGTA, 2 mM MgCl2, 10 mM HEPES, 2 mM Mg + -ATP, 0.2 mM Na + -GTP. The protocol consisted of a set of 15 depolarizing steps lasting 400 ms (Δ + 10 pA, starting at –20 pA). The recordings were conducted before (baseline) and during a drug application. The BK channel blocker paxilline (300 nM) and ethanol (50 mM) were bath applied for 8–12 min and 6–8 min, respectively. The recorded IVs were analyzed by NeuroExpress 19.0 [28]. Two cells (one WT ethanol and one KI ethanol) were excluded from the analysis because their data met Grubbs’ outlier criterion [29].

### Tremors

Tremors were scored according to the following scale [30]: 0 = no tremor; 1 = no resting tremor, short-lasting low-intensity shaking elicited by handling; 2 = no resting tremor, continuous low-intensity shaking elicited by handling; 3 = spontaneous low-intensity tremor, aggravated by handling; 4 = severe spontaneous tremor, convulsive episode elicited by handling; score 5 was not observed.

### Ethanol clearance rate

Mice were i.p. injected with 2 g/kg ethanol (20% v:v, 0.13 mL/10 g body weight). Tail vein blood was collected 30 min, 90 min and 180 min later and processed for blood ethanol concentration (BEC) determination by gas chromatography and flame ionization detection (Agilent 7820A).

### Motor coordination and ethanol-induced ataxia

Motor coordination was evaluated using an AccuRotor rotarod (Accuscan Instruments) accelerating from 4 to 40 rpm over 300 s. Mice were positioned on the rotating rod and speed at fall (rpm) was recorded. For motor learning, mice were subjected to 5 trials per day (30–90 min apart) for 3 consecutive days. For ataxia testing, the rod was rotating at a constant speed of 8 rpm and the mice had to stay on the rod for at least 30 s to pass. Ataxia testing was conducted 4-5 days after the last training trial and all mice were able to pass the criterion. They were then i.p. injected with 1.5 g/kg ethanol (0.1 mL/10 g body weight) and tested approximately every 4 min until they were able to pass the criterion again. At this point, blood was collected from the retroorbital sinus and processed for BEC determination using a GM7 analyzer (Analox Instruments).

### Ethanol-induced sedation and hypothermia

Baseline body temperatures were first determined using a MicroTherma 2 K thermometer (ThermoWorks) fitted with a rectal probe. Mice were then i.p. injected with 3.5 g/kg ethanol (0.2 mL/10 g body weight), which resulted in loss of righting response (i.e., sedation). Mice were placed on their backs and the time at which each mouse regained its righting response was recorded. At this point, retroorbital blood was sampled and BECs were determined using a GM7 analyzer. Body temperatures were again recorded 60 and 120 min after injection.

### Ethanol-induced analgesia

A digital Randall-Selitto apparatus (Harvard Apparatus 76-0234) was used to measure mechanical nociceptive thresholds, as described in [31]. The mouse was habituated to enter a restrainer made of woven wire (stainless steel 304 L 200 mesh, Shanghai YiKai) over the course of 3 days. On testing days, the mouse was gently introduced into the restrainer and the distal portion of the tail was positioned under the conic tip of the apparatus. The foot switch was then depressed to apply uniformly increasing pressure onto the tail until the first nociceptive response (struggling or squeaking) occurred. The force (in g) eliciting the nociceptive response was recorded. A cutoff force of 600 g was enforced to prevent tissue damage. The measure was repeated on the medial and proximal parts of the tail of the same mouse, with at least 30 seconds between each measure. The average of the three measures (distal, medial, proximal) was used as nociceptive value for that day. The analgesic effect of ethanol was tested over 4 consecutive days using a Latin square design. Testing was conducted 5 min after i.p. injection of 20% v:v ethanol (0, 1.5, 2 and 2.5 g/kg, 0.1-0.17 mL/10 g body weight).

### Ethanol-conditioned place preference

The apparatus was made of matte black acrylic and consisted of a 42 cm long x 21 cm wide x 31 cm high rectangular box (inner dimensions) with a removable central divider (ePlastics, San Diego). In one compartment, the floor was covered with coarse mesh (stainless steel 304 L 10 mesh, Shanghai YiKai) and the walls were decorated with white discs (5-cm dot sticker, ChromaLabel). In the other compartment, the floor was smooth and the walls were uniformly black. Pre-conditioning, conditioning, and post-conditioning trials were conducted on consecutive days, 2 h into the light phase of the circadian cycle. During the pre-conditioning and post-conditioning tests, mice had access to both compartments during 15 min and their motion was video-recorded by a ceiling-mounted camera connected to ANY-maze (Stoelting Co.). During the conditioning trials, the mice were i.p. injected with saline or 2 g/kg ethanol (20% v:v, 0.13 mL/10 g body weight) and immediately confined to the compartment paired with this treatment during 30 min. A biased design was used to assign compartments to saline or ethanol for each mouse, i.e., ethanol was always assigned to the least favorite compartment (mesh floor for 6 WT and 9 KI mice, smooth floor for 5 WT and 6 KI mice). Treatments were alternated for a total of 8 conditioning trials (4 saline and 4 ethanol) and the order of treatment was counterbalanced within each genotype. Conditioned place preference was reflected by an increase in the time spent in the ethanol-paired compartment after *vs*. before conditioning.

### Alcohol drinking

Mice were single-housed 3 days before testing started and remained single-housed throughout the duration of all drinking experiments. Voluntary alcohol consumption was assessed as a two-bottle choice (2BC) between water and ethanol in the home cage. Ethanol concentration was 15% v:v (as in [32] and [33]), except for the continuous/intermittent access repeat experiment, which used 20% w:v (as in [34]). For limited access, 2-h 2BC sessions were started at the beginning of the dark phase (except for the penitrem A study, in which sessions were started 2 h into the dark phase) and conducted Mon-Fri. For continuous access, bottles were weighed daily 3 h into the dark phase. For intermittent access, the ethanol bottle was present for 24-h periods starting 3 h into the dark phase on Mon, Wed, and Fri, and replaced with a second water bottle the rest of the time. In all paradigms, the positions of the ethanol and water bottles were alternated each day to control for side preference. Ethanol intake was determined by weighing bottles before and after the session, subtracting the weight lost in bottles placed in an empty cage (to control for spill/evaporation) and dividing by the mouse bodyweight (measured weekly). A similar procedure was used to assess 2-h saccharin (0.005% w:v) consumption in the penitrem A study. Ethanol intake is expressed as g of ethanol molecule, while saccharin intake is expressed as g of saccharin solution; both are normalized to the mouse’s body weight.

CIE vapor inhalation was used to increase voluntary ethanol intake in 2-h 2BC sessions, as described in [13, 32]. Mice were first subjected to two weeks of 2BC (Mon-Fri) and split into two groups of equivalent baseline ethanol intake (average of last three days). Weeks of CIE (or air) inhalation (4 × 16-h intoxication/8-h withdrawal, Mon-Fri) were then alternated with weeks of 2BC (Mon-Fri) for a total of 3–5 rounds.

BK channel modulators were injected 30 min prior to 2-h 2BC.

### Chronic intermittent ethanol (CIE) vapor inhalation

The inhalation chambers were made of sealed plastic mouse cages (Allentown). An electronic metering pump (Iwaki EZB11D1-PC) dripped 95% ethanol into a flask placed on a warming tray at a temperature of 50°C. Drip rate was adjusted to achieve target BECs of 150-250 mg/dL. An air pump (Hakko HK-80L) conveyed vaporized ethanol from the flask to each individual chamber. The air flow was set at a rate of 15 L/min for each pair of chambers. Each chamber was diagonally divided by a mesh partition to provide single housing for two mice. Mice were injected i.p. with ethanol (1.5 g/kg) and pyrazole (68 mg/kg, Sigma-Aldrich, P56607) diluted in saline, in a volume of 0.1 mL/10 g body weight, before each 16-h ethanol vapor inhalation session. Blood was sampled from the caudal vein at the end of a 16-h intoxication session. The tip of the tail was nicked with a scalpel blade, blood was collected with a heparinized capillary tube and centrifuged at 13,000 g for 10 min. BECs were measured using a GM7 analyzer or by gas chromatography and flame ionization detection. On CIE weeks, control (air) mice received pyrazole only. In our experience, mice that do not consume alcohol prior to CIE exposure typically show higher sensitivity to vapor inhalation (risk of overexposure). Therefore, to promote survival in experiments involving CIE without 2BC, we used milder parameters of vapor exposure than for CIE-2BC experiments. Average BECs in each CIE-exposed cohort are reported in Table 1.

**Table 1:** Average blood ethanol concentrations (BEC) during chronic intermittent ethanol (CIE) vapor inhalation.

Experiment	Average BEC (mg/dL)
Penitrem A CIE-2BC (Fig. 1D)	278.8 ± 15.3
Paxilline CIE-2BC (Fig. 1E)	223.3 ± 11.0
BMS-204352 CIE-2BC (Fig. 1F)	211.0 ± 16.9
K361N CIE-2BC Experiment #1 (Fig. 5B)	WT: 184.4 ± 24.0 KI: 176.0 ± 25.9
K361N CIE-2BC Experiment #2 (Figs. 5D and 5F)	WT males: 232.6 ± 25.6 KI males: 250.2 ± 23.5 WT females: 214.0 ± 21.7 KI females: 233.4 ± 15.0
K361N echoMRI/CLAMS (Fig. 7A-D)	Week 1: WT: 46.2 ± 3.4 KI: 46.0 ± 10.3 Weeks 2-5: WT: 156.0 ± 9.6 KI: 127.4 ± 3.4
K361N locomotor activity (Fig. 7E)	WT: 132.2 ± 8.5 KI: 129.7 ± 9.3

### Metabolism and actimetry

Mice were exposed to CIE every other week, starting with a priming week at sub-intoxicating BECs, and followed by 4 weeks at intoxicating BECs. Body composition was analyzed by quantitative nuclear magnetic resonance (EchoMRI 3-in-1, EchoMRI LLC) 72 h after the last vapor exposure. Mice were then immediately placed in individual metabolic cages (Comprehensive Laboratory Animal Monitoring System [CLAMS], Oxymax, Columbus Instruments), at the beginning of the dark phase. The following data were collected every 18 min for a total of 108 h: oxygen consumption (VO_2_), carbon dioxide production (VCO_2_), food intake, water intake, and locomotor activity. The respiratory exchange ratio (RER), calculated as VCO_2_/VO_2_, provides an indicator of the substrate being metabolized, ranging from 0.7 when the predominant fuel source is fat to 1 when the predominant fuel source is carbohydrate [35]. Locomotor activity counts (beam interruptions) were used by CLAMS-HC Sleep Detection function to track sleeping bouts, as defined by 4 (or more) consecutive 10-sec epochs with 0 activity counts [36]. The first 12 hours (dark phase) were considered habituation and excluded from analysis. The following 96 h were binned by 12-h light and dark phases and averaged across the 4 days for statistical analysis. It should be noted that polysomnography would have been more appropriate than actimetry for sleep measurement. One mouse (KI, air) was excluded from analysis of body composition and metabolic measures because its data met Grubbs’ outlier criterion [29].

### Circadian rhythmicity

Mice were exposed to CIE every other week for a total of 4 weeks and transferred to individual locomotor activity cages (Photobeam Activity System-Home Cage, San Diego Instruments) 72 h after the last vapor exposure. Mice were maintained on a 12 h/12 h light/dark cycle for 7 consecutive days, then switched to constant darkness for an additional 11 days. Ambulation counts represent consecutive beam breaks (8 × 4 beams in the 18.5” x 10” frame) and were collected in 1-h bins. Chi-square periodogram analysis was conducted in R (‘zeitgebr’ package, https://github.com/rethomics/zeitgebr) to determine the circadian period length and relative power during constant darkness [37, 38], using the last 240 hours of recording and a 6-min resampling rate.

### Statistical analysis

Data were analyzed in Prism 9.5.1 (GraphPad) and Statistica 13.3 (Tibco Software Inc.). Electrophysiological data were analyzed by multiple paired t-tests using a false discovery rate of 1%. Tremor scores were analyzed by Kruskal-Wallis analysis of variance (ANOVA) of the area under the curve, followed by Dunn’s comparisons to vehicle. Alcohol and saccharin drinking was analyzed by repeated-measures (RM) ANOVA with dose (pharmacological experiments), week (2-h 2BC acquisition and escalation, weekly averages), or access schedule (continuous/intermittent 2BC, last 24-h period of each phase) as within-subject variable. *Posthoc* tests were conducted using Dunnett’s test for comparisons to vehicle, and Šidák’s test otherwise. For escalation, significant week x vapor interactions were followed up with two-way ANOVAs (genotype, vapor) on individual weeks. The effect of genotype on body weight, ataxia, and sedation was analyzed by one-way ANOVA. Ethanol’s clearance rate, hypothermia, analgesia, and place preference were analyzed by two-way RM-ANOVA, with time, dose, or conditioning as within-subject variable and genotype as between-subject variable. EchoMRI data, circadian period length and relative power were analyzed by two-way ANOVA (genotype, vapor). CLAMS data were analyzed by three-way RM-ANOVA, with phase as within-subject variable and genotype and vapor as between-subject variables. When there was a significant interaction between phase and vapor, two-way ANOVAs were further conducted for each phase. The Greenhouse-Geisser correction was used to adjust for lack of sphericity in RM-ANOVAs with three or more levels. Data are expressed as mean ± s.e.m.

## Results

### Non-tremorgenic pharmacological inhibition of BK channel activity can modulate moderate alcohol drinking

We first sought to examine the contribution of BK channels to voluntary ethanol consumption using a pharmacological approach in C57BL/6 J males. Since ethanol can activate some neuronal BK channels [1–3, 12, 39], we hypothesized that blocking BK channels may interfere with the motivational properties of ethanol and increase (to overcome BK channel blockade) or decrease (if blockade is unsurmountable) alcohol drinking. On the other hand, in central amygdala GABAergic synapses, ethanol can mimic the effect of BK blockers [40], such that BK channel inhibitors may prime alcohol seeking at low doses and substitute for ethanol (i.e., reduce the motivation to consume alcohol) at higher doses.

**Fig. 1:**
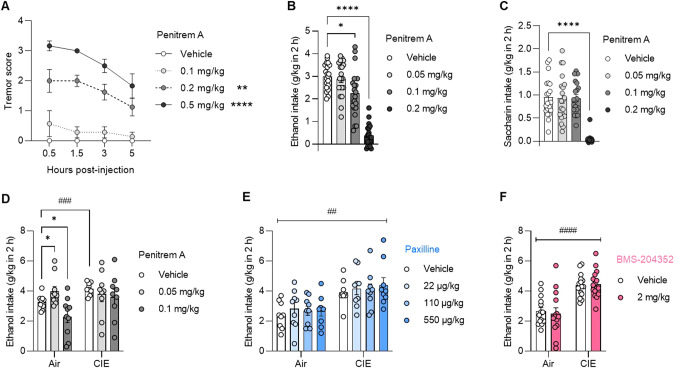
Effects of BK channel pharmacological modulators on baseline and escalated alcohol drinking. **A**. Penitrem A, a BK channel blocker, induced tremors in a dose-dependent manner in alcohol-naïve C57BL/6J males (between-subject design). Error bars show s.e.m. C57BL/6J male mice were given access to voluntary alcohol (**B**, **D**–**F**) or saccharin (**C**) consumption in 2-h two-bottle choice (2BC) sessions. The BK channel blockers penitrem A (**B**–**D**) or paxilline (**E**) or the BK channel opener BMS-204352 (**F**) were injected i.p. 30 min before 2BC (within-subject design). Some mice were exposed to chronic intermittent ethanol (CIE) vapor inhalation to increase voluntary ethanol intake, compared to mice inhaling air only (between-subject design). Significant difference with vehicle: *p < 0.05; **; ****p < 0.0001. Significant effect of CIE: ^##^*p* < 0.01; ^###^*p* < 0.001; ^####^*p* < 0.0001.

We first used penitrem A, a brain-penetrant fungal alkaloid that potently inhibits BK channels [41, 42]. Penitrem A induced tremors in a dose-dependent manner (Fig. 1A, main effect of dose: H_3,24_ = 23.4, *p* < 0.0001; *posthoc* comparisons to vehicle: 0.2 mg/kg, *p* = 0.0055; 0.5 mg/kg, *p* < 0.0001), as reported previously [43]. Consistent with its tremorgenic effect, the dose of 0.2 mg/kg abolished both ethanol (Fig. 1B, dose effect: F_1.899,36.08_ = 65.5, *p* < 0.0001; vehicle *vs*. 0.2 mg/kg, *p* < 0.0001) and saccharin (Fig. 1C, dose effect: F_1.864,35.41_ = 59.6, *p* < 0.0001; vehicle *vs*. 0.2 mg/kg, *p* < 0.0001) drinking. The dose of 0.1 mg/kg reduced ethanol intake (*p* = 0.0169) without affecting saccharin intake (*p* = 0.98). The lowest dose of 0.05 m/kg did not affect ethanol (*p* = 0.57) or saccharin (*p* = 0.98) intake (Fig. 1B-C).

Based on our previous findings in BK β1 and β4 knockout (KO) mice [13], we reasoned that the effect of BK channel modulation may be sensitive to CIE exposure. There was a significant interaction between vapor and penitrem A (F_2,34_ = 3.47, *p* = 0.0426) whereby vehicle-injected CIE mice consumed more alcohol than their air-exposed counterparts (*p* = 0.0008) and penitrem A had dose-dependent effects in air mice (increase at 0.05 mg/kg, *p* = 0.0165; decrease at 0.1 mg/kg, *p* = 0.0355) but not in CIE mice (Fig. 1D).

Tremorgenic mycotoxins can inhibit BK channels via different mechanisms and may therefore have a differential effect on ethanol-induced potentiation of BK-mediated currents. Notably, the association of β1 subunits reduces BK channel sensitivity to penitrem A by 10-fold, while it does not affect sensitivity to paxilline, a highly selective BK channel blocker [42, 44]. Since β1 subunits influence ethanol intake escalation in CIE-exposed mice [13], we next tested the effect of paxilline in both air and CIE mice. We limited our analysis to non-tremorgenic doses (see *Methods* for dose range determination). Paxilline did not affect ethanol intake regardless of vapor exposure (Fig. 1E, dose effect: F_1.865,29.84_ = 1.0, *p* = 0.38; vapor effect: F_1,16_ = 11.2, *p* = 0.004; dose x vapor interaction: F_3,48_ = 0.27, *p* = 0.85).

To further investigate the ability of BK channels to modulate ethanol intake, we tested the effect of a BK channel opener, BMS-204352. At 2 mg/kg, a dose that rescues several behavioral deficits of *Fmr1* KO mice [17, 18], BMS-204532 did not impact moderate (air) or excessive (CIE) ethanol drinking (Fig. 1E, treatment effect: F_1,28_ = 0.09, *p* = 0.77; vapor effect: F_1,28_ = 28.3, *p* < 0.0001; treatment x vapor interaction: F_1,28_ = 0.6, *p* = 0.43).

### Generation and validation of BK α K361N knockin mice

The significance of pharmacological manipulations is inherently limited because they perturb the physiological activity of BK channels rather than selectively targeting their ethanol-sensing capacity. We therefore turned to a genetic approach to probe the role of ethanol’s direct action at BK channels in the motivation to consume alcohol. An asparagine substitution of residue K361 of the mouse BK α subunit was shown to abolish ethanol’s ability to increase BK channel steady-state activity without affecting unitary conductance, calcium sensitivity, or voltage sensitivity, thereby providing a unique opportunity to selectively disrupt the direct effect of ethanol on BK channels [15].

**Fig. 2:**
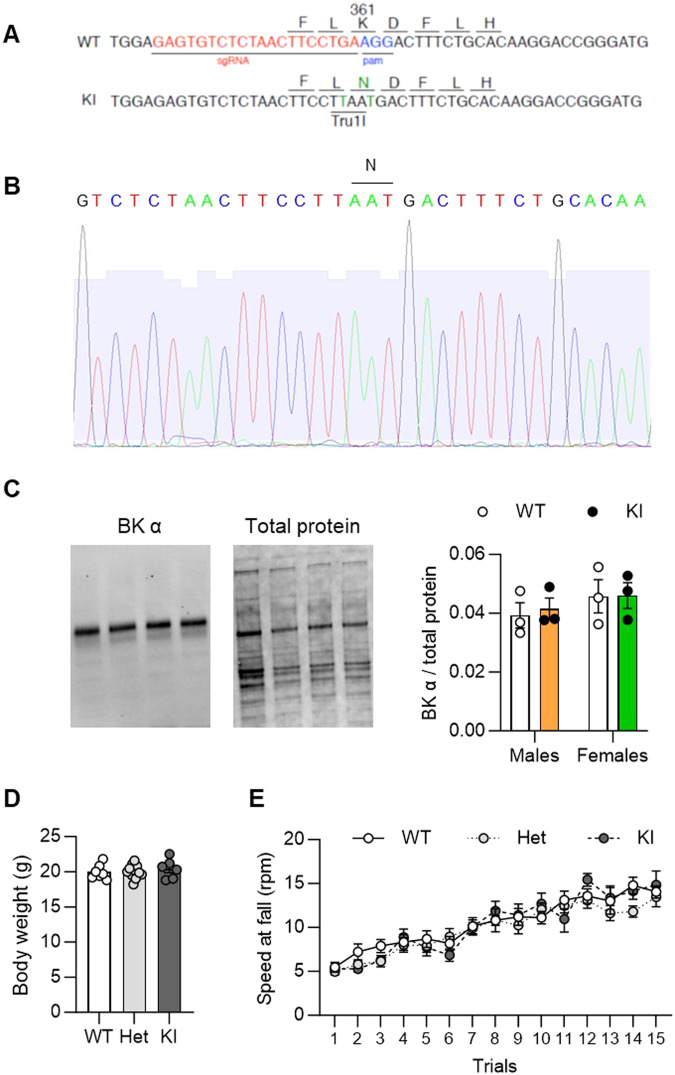
Generation of BK α K361N knockin mice. **A**. Design of the CRISPR/Cas9 construct used to introduce the K361N substitution in C57BL/6J mice. The single guide RNA (sgRNA) sequence is shown in red, the protospacer adjacent motif (PAM) is shown in blue, and the two mutated nucleotides are shown in green. WT, wildtype allele; KI, knockin allele. **B** Verification of the mutated sequence in cDNA prepared from the brain of a K361N KI mouse. The triplet encoding the K361N substitution is highlighted. **C** Quantification of BK α protein levels in brain membranes from adult males and females. The immunoblotting signal was normalized to total protein transferred. Representative images are shown, uncropped blots and molecular weight markers are provided in Supplementary Fig. 1. **D** Body weights measured in males at 6 weeks of age. **E** Motor coordination measured in the accelerating rotarod assay in adult males. Error bars show s.e.m. There was no effect of genotype on either measure.

Accordingly, we generated a knockin (KI) mouse expressing the K361N mutant instead of the wildtype (WT) BK α on a C57BL/6J background. A CRISPR/Cas9 strategy was used to introduce two nucleotide mutations in the *Kcnma1* gene: A G-to-T missense mutation modifying the triplet encoding K361 into an asparagine-coding triplet, and a silent G-to-T mutation introducing a Tru1I restriction site to facilitate mouse genotyping (Fig. 2A). KI mice were viable, and all three genotypes (KI, Het, and WT) were obtained in Mendelian proportions. The presence of the mutations in the *Kcnma1* mRNA was confirmed by mouse brain cDNA sequencing (Fig. 2B). The K361N substitution did not alter BK α protein levels in brain membrane fractions from adult males and females (Fig. 2C and Supplementary Fig. 1, effect of genotype, F_1,8_ = 0.08, *p* = 0.79; effect of sex, F_1,8_ = 1.4, *p* = 0.26; interaction, F_1,8_ = 0.05, *p* = 0.83). We verified that the basal function of BK channels was preserved in KI mice, based on the known phenotype of mice missing BK α. Accordingly, while BK α KO mice displayed 15-20% smaller body weights than their WT counterparts at 4 and 8 weeks of age [45], there was no effect of the K/N361 genotype on body weight at 6 weeks of age (Fig. 2D, F_2,22_ = 0.4, *p* = 0.70). Furthermore, while BK α KO mice displayed major motor coordination deficits [45], BK α K361N KI mice acquired the accelerating rotarod task at the same rate as their Het and WT counterparts (Fig. 2E, effect of trial: F_14,336_ = 37.2, *p* < 0.0001; effect of genotype: F_2,24_ = 0.8, *p* = 0.48; trial x genotype interaction: F_28,336_ = 0.8, *p* = 0.73).

**Fig. 3:**
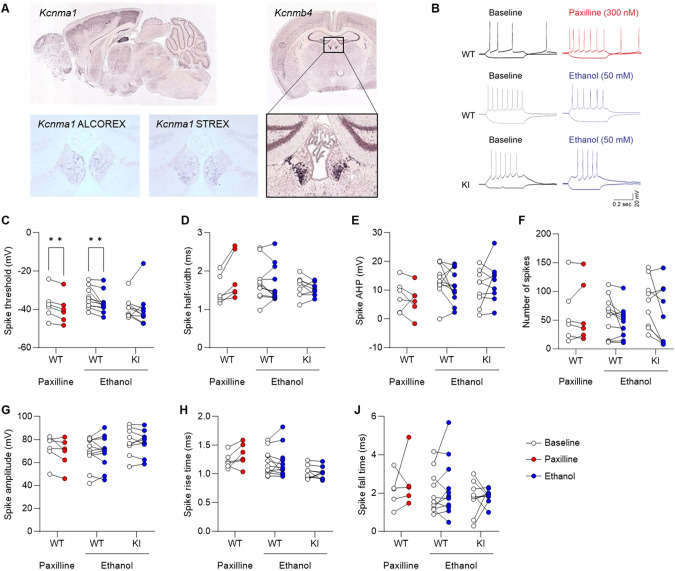
Electrophysiological validation of BK α K361N knockin mice. **A**. Distribution of transcripts encoding BK channel α (*Kcnma1*) and β4 (*Kcnmb4*) (Allen Mouse Brain Atlas, https://mouse.brain-map.org, experiments 74578206 and 72283793), as well as alternatively spliced *Kcnma1* exons ALCOREX and STREX, in mouse brain sections, showing their enrichment in the medial habenula (mHb). **B** Representative traces from electrophysiological recordings of mHb neurons from WT and KI brain slices before (open circles in **C**–**J**) and during (closed circles in **C**–**J**) treatment with paxilline (300 nM, red) or ethanol (50 mM, blue). **C** Paxilline and ethanol both reduced the spike threshold in WT neurons, but KI neurons were insensitive to ethanol. There was no significant effect of paxilline or ethanol on spike half-width (**D**), spike afterhyperpolarization (**E**), spike number (**F**), spike amplitude (**G**), spike rise time (**H**), spike fall time (**J**). Baseline vs. drug: **q < 0.01 (FDR 1%).

We next sought to confirm that the K361N substitution imparts ethanol resistance to native BK channels, as seen with recombinant channels expressed in *Xenopus* oocytes. Electrophysiological recordings were obtained from the medial habenula (mHb), a brain region that expresses high levels of BK channel subunits (*Kcnma1* and *Kcnmb4* transcripts), including an alternatively spliced *Kcnma1* exon known to confer sensitivity to ethanol (ALCOREX, [46]) (Fig. 3A). To identify firing parameters that are modulated by BK channels in mHb neurons, the effect of paxilline (300 nM) was first tested in WT slices. Paxilline significantly reduced spike threshold (*q* = 0.0019), tended to increase spike half-width (*q* = 0.065), and did not affect spike afterhyperpolarization, number, amplitude, rise time, or fall time (q’s > 0.36, Fig. 3C–J). The effect of ethanol (50 mM) was then tested in both WT and KI slices. Ethanol reduced spike threshold in WT (*q* = 0.0091) but not in KI (*q* = 0.45) cells (Fig. 3C). Ethanol did not significantly affect any of the other parameters in either WT or KI cells (*q*’s > 0.17, Fig. 3D-J). In conclusion, ethanol mimics BK channel blockade in mHb neurons and this effect is abolished by the K361N substitution.

### The BK α K361N substitution does not affect sensitivity to acute effects of alcohol in vivo

**Fig. 4:**
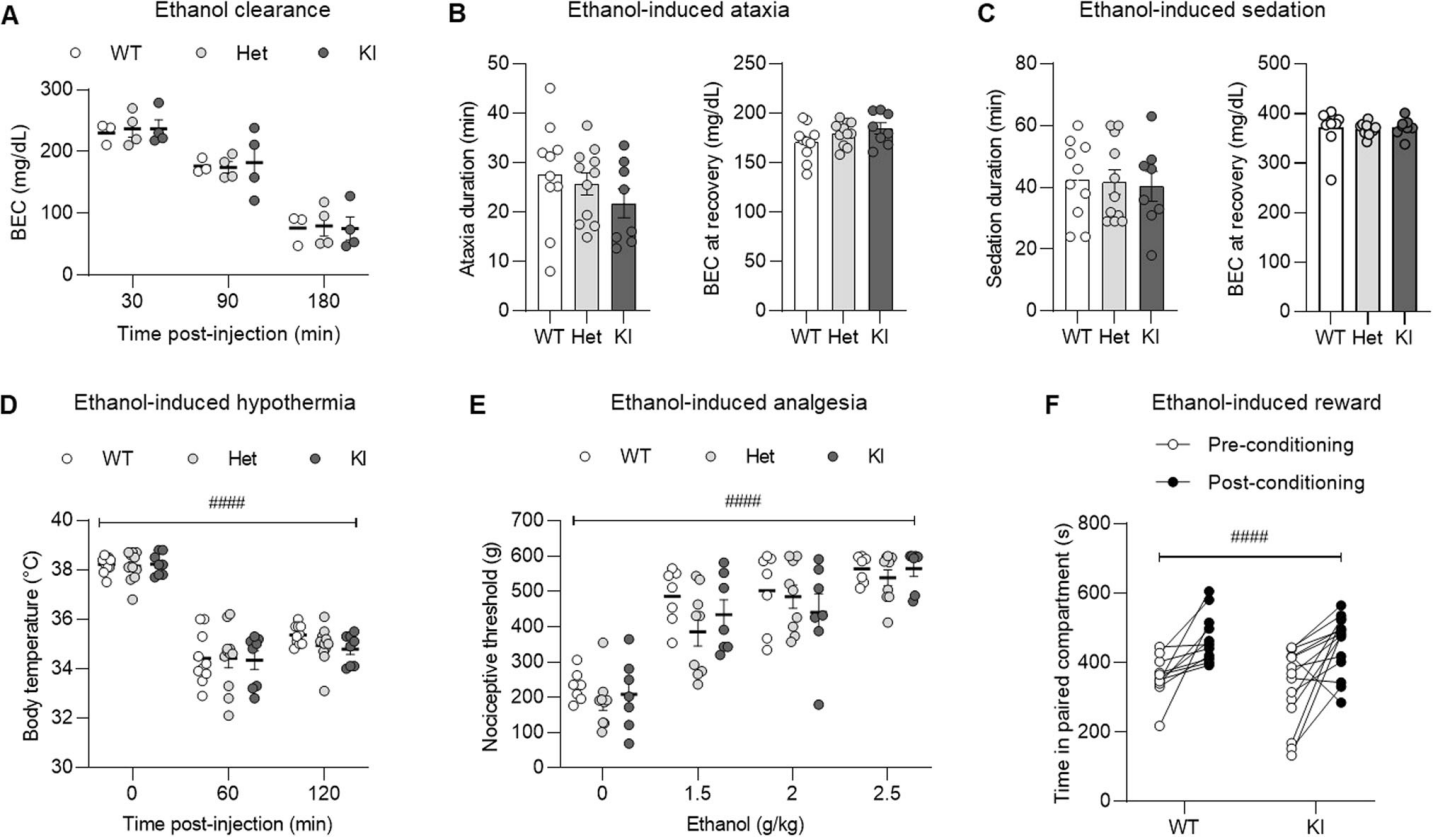
Effect of the BK α K361N substitution on acute alcohol sensitivity in vivo. Measures of alcohol metabolism and intoxication were obtained in BK α K361N WT, Het and KI males acutely exposed to ethanol (i.p.). **A**. Blood ethanol concentration (BEC) clearance time-course.Ethanol-induced ataxia (**B**, fixed-speed rotarod), sedation (**C**, loss of righting response), hypothermia (**D**), analgesia (**E**, tail pressure test) and reward (**F**, conditioned place preference). Main effect of ethanol: ^###^*p* < 0.001. None of the measures was significantly affected by genotype.

We first verified that the mutation did not alter the clearance rate of ethanol (effect of time: F_1.202,9.616_ = 359.6, *p* < 0.0001; effect of genotype: F_2,8_ = 0.01, *p* = 0.99; time x genotype interaction: F_4,16_ = 0.2, *p* = 0.91, Fig. 4A). In the rotarod assay, WT, Het, and KI males were similarly sensitive to the loss of motor coordination induced by 1.5 g/kg ethanol; there was no effect of genotype on ataxia duration (F_2,26_ = 1.0, *p* = 0.38) and on BECs measured at recovery (F_2,26_ = 2.0, *p* = 0.16) (Fig. 4B). Likewise, WT, Het and KI males exhibited similar durations of loss-of-righting-response following administration of 3.5 g/kg ethanol (F_2,26_ = 0.5, *p* = 0.95) and similar BECs at recovery (F_2,26_ = 0.06, *p* = 0.94) (Fig. 4C). The amplitude of hypothermia was also identical across genotypes (effect of time: F_1.666,43.31_ = 239.6, *p* < 0.0001; effect of genotype: F_2,26_ = 0.4, *p* = 0.66; time x genotype interaction: F_4,52_ = 0.5, *p* = 0.71, Fig. 4D). Ethanol exerted similar analgesic effects in WT, Het and KI males at 1.5–2.5 g/kg doses (effect of dose: F_2.249,44.98_ = 61.0, *p* < 0.0001; effect of genotype: F_2,20_ = 2.0, *p* = 0.16; dose x genotype interaction: F_6,60_ = 0.6, *p* = 0.73, Fig. 4E). Finally, the rewarding effect of 2 g/kg ethanol was equivalent in WT and KI males, as measured by conditioned place preference (effect of conditioning: F_1,24_ = 25.6, *p* < 0.0001; effect of genotype: F_1,24_ = 0.6, *p* = 0.43; conditioning x genotype interaction: F_1,24_ = 0.04, *p* = 0.84, Fig. 4F). There was no effect of genotype or conditioning on the distance traveled in the apparatus (Supplementary Fig. 2). Altogether, we did not detect any influence of the BK α K361N substitution on the sensitivity of male mice to multiple behavioral and physiological acute effects of moderate and high doses of ethanol.

### The BK α K361N substitution does not reproducibly alter alcohol drinking under limited, continuous, or intermittent access

**Fig. 5:**
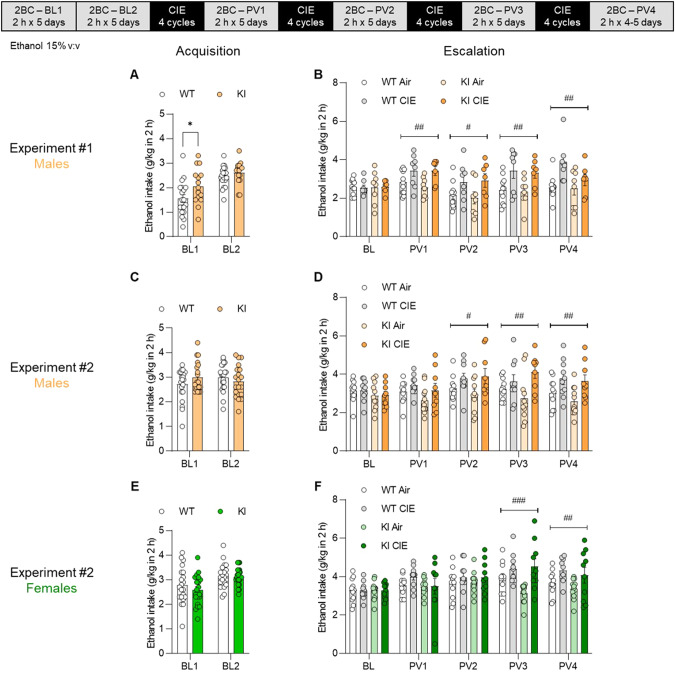
Effect of the BK α K361N substitution on baseline and escalated alcohol drinking in the CIE-2BC model. BK α K361N WT and KI mice were given access to voluntary alcohol consumption in 2-h two-bottle choice sessions prior to (Acquisition, **A**, **C**, **E**) and in-between (Escalation, **B**, **D**, **F**) weeks of chronic intermittent ethanol (CIE) vapor inhalation. A first experiment was conducted in males only (**A**, **B**) and a repeat experiment included both males (**C**, **D**) and females (**E**, **F**). Weekly averages are shown (daily values are provided in Supplementary Fig. 3). *p < 0.05, WT *vs*. KI. Main effect of vapor: ^#^*p* < 0.05; ^##^*p* < 0.01; ^###^*p* < 0.001.

A first group of mice (all males) was given access to voluntary alcohol consumption in 2-h 2BC sessions. The KI males consumed more alcohol than their WT counterparts during the first baseline (BL) week, but the difference subsided by the second week, with the two genotypes stabilizing at similar levels (main effect of genotype, F_1,32_ = 3.5, *p* = 0.069; *posthoc* on BL1, *p* = 0.032; BL2, *p* = 0.75; Fig. 5A and Supplementary Fig. 3A). Half of the mice were then exposed to weeks of CIE to trigger voluntary intake escalation during intercalated weeks of 2BC drinking [32] (Fig. 5B and Supplementary Fig. 3B). As expected, there was a significant week x vapor interaction (F_4,120_ = 5.1, *p* = 0.0008), reflecting the escalation of voluntary alcohol consumption in CIE mice but not air mice. A significant main effect of vapor was detected on each post-vapor (PV) week (F_1,30_ > 7.0, *p* < 0.02), along with a trend for genotype effect on PV4 (F_1,30_ = 2.9, *p* = 0.097), whereby KI mice consumed less alcohol than WT mice. To follow up on the BL1 and PV4 observations, we repeated the experiment and included both sexes (Fig. 5C-F and Supplementary Fig. 3C-F). In this second experiment, there was no effect of genotype or trend thereof on any of the baseline or post-vapor weeks. A significant main effect of vapor was detected at PV2, PV3, and PV4 in males (F_1,37_ > 6.9, *p* < 0.02), and PV3 and PV4 in females (F_1,39_ > 7.5, *p* < 0.01).

**Fig. 6:**
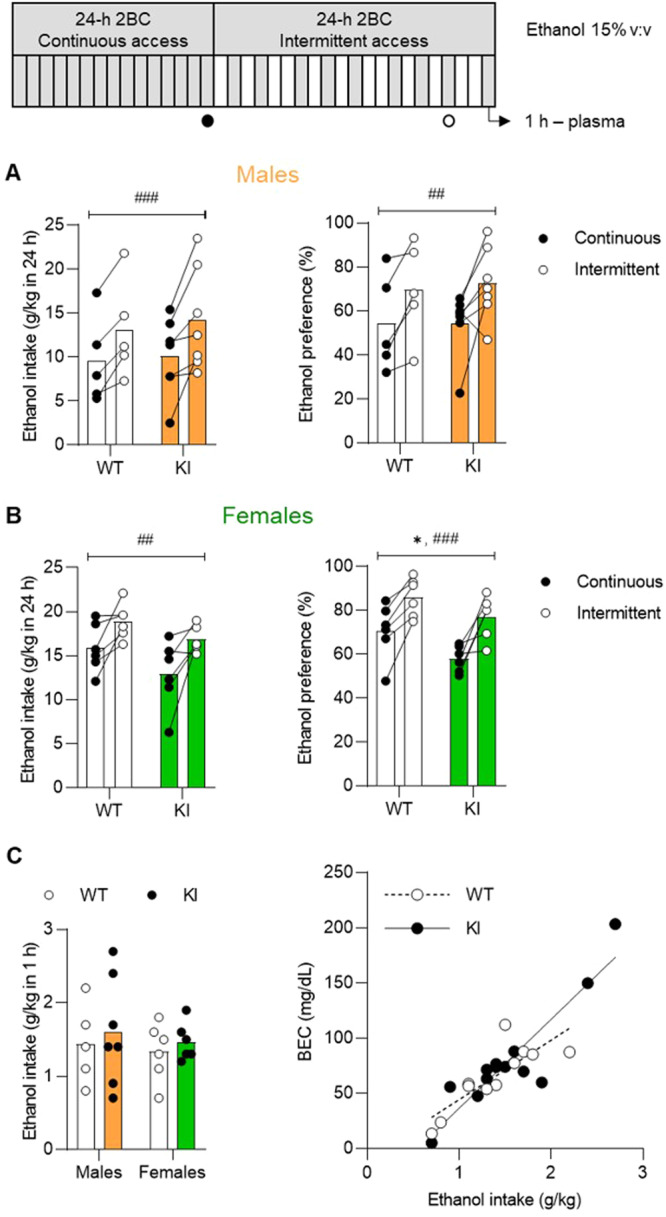
Effect of the BK α K361N substitution on alcohol drinking under continuous and intermittent access. BK α K361N WT and KI males (**A**) and females (**B**) were given continuous access to voluntary alcohol consumption (two-bottle choice) before switching to an intermittent schedule of access (24 h, three times per week). Ethanol intake (left) and preference (right) are shown for the last 24-h period of each phase (daily values are provided in Supplementary Fig. 4). Main effect of access schedule: ^##^*p* < 0.01; ^###^*p* < 0.001. Main effect of genotype: *p < 0.05. **C** On the last day of intermittent access, blood ethanol concentrations were measured 1 h after alcohol introduction to verify correlation with intake.

BK α WT and K361N KI mice were then tested in another model of alcohol drinking escalation in which access to alcohol switches from continuous to intermittent for 24-h periods [33, 34]. As expected, intermittent access significantly increased both ethanol intake and ethanol preference in both males (intake: F_1,10_ = 22.9, *p* = 0.0007; preference: F_1,10_ = 12.8, *p* = 0.0050, Fig. 6A and Supplementary Fig. 4A, B) and females (intake: F_1,10_ = 14.9, p = 0.0032; preference: F_1,10_ = 32.3, *p* = 0.0002, Fig. 6B and Supplementary Fig. 4C, D). In addition, a significant main effect of genotype was detected in females, whereby KI mice had a lower preference for ethanol than their WT counterparts (F_1,10_ = 5.4, *p* = 0.042, Fig. 6B). Finally, there was no effect of genotype on ethanol intake and BECs measured 1 h after alcohol access resumption (Fig. 6C). As expected, BECs correlated significantly with intake in both genotypes (WT: R^2^ = 0.68, *p* = 0.0019; KI: R^2^ = 0.83, *p* < 0.0001). To follow up on the preference phenotype, we repeated the experiment in a separate group of females. Intermittent access again increased ethanol intake (F_1,15_ = 28.8, *p* < 0.0001) and preference (F_1,15_ = 19.4, *p* = 0.0005), but there was no effect of genotype or trend thereof on either measure (Supplementary Fig. 5).

In conclusion, the BK α K361N substitution does not reliably affect alcohol drinking under multiple modalities of moderate and excessive intake.

### The BK α K361N substitution does not impact the effects of CIE on metabolism, food intake, and locomotor activity

**Fig. 7:**
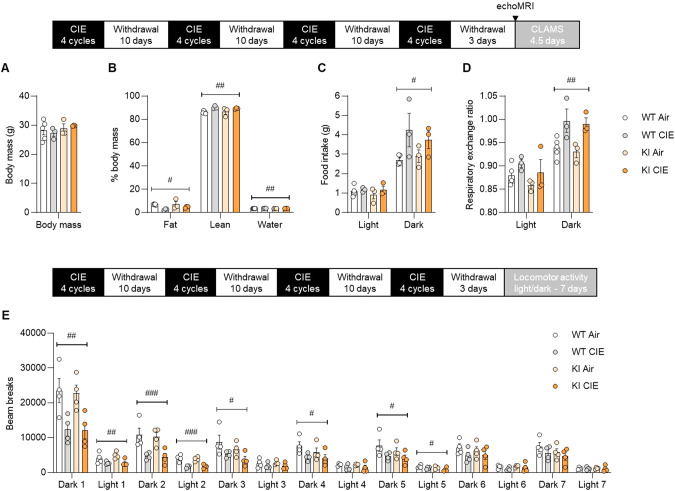
Effect of the BK α K361N substitution on the metabolic and locomotor effects of chronic intermittent ethanol (CIE) exposure. BK α K361N WT and KI males were exposed to air or chronic intermittent ethanol (CIE) vapor inhalation. In a first experiment, body mass (**A**) and body composition (**B**) were determined 3 days into withdrawal. Food intake (**C**) and respiratory exchange ratio (**D**) were then recorded in metabolic chambers for 4.5 days. In a second experiment, locomotor activity was recorded starting 3 days into withdrawal (**E**). Main effect of vapor: ^#^*p* < 0.05; ^##^*p* < 0.01; ^###^*p* < 0.001. None of the measures was significantly affected by genotype.

We then tested whether KI mice might exhibit differential sensitivity to other consequences of CIE exposure relevant to AUD, such as metabolic [47] and sleep [48] disturbances. EchoMRI analysis indicated that CIE significantly altered body composition, reducing fat content (F_1,10_ = 9.8, *p* = 0.011) while increasing lean (F_1,10_ = 10.6, *p* = 0.0086) and water (F_1,10_ = 13.8, *p* = 0.0040) content, in the absence of body weight change (F_1,10_ = 0.001, *p* = 0.98, Fig. 7A, B). Metabolic monitoring also revealed increases in dark-phase food intake (F_1,10_ = 7.3, *p* = 0.023, Fig. 7C) and dark-phase RER (F_1,10_ = 15.7, *p* = 0.0027, Fig. 7D) in CIE-withdrawn mice. The K361N substitution did not influence any of these outcomes (F’s < 1.0, *p*’s > 0.34 for main effect of genotype and genotype x vapor interaction). Furthermore, neither genotype nor CIE affected actimetry-based sleep measures (Supplementary Fig. 6).

Finally, given the role of BK channels in regulating neuronal excitability in the suprachiasmatic nucleus (the primary circadian pacemaker in mammals) [49, 50] and the desynchronization of biological rhythms observed in AUD [51, 52], we sought to determine whether the action of ethanol on BK channels could be responsible for a disruption of circadian rhythmicity in CIE-exposed mice. Under a standard light-dark cycle, the ambulation of CIE-exposed mice was significantly reduced up to withdrawal day 8 (vapor x time interaction: F_13,156_ = 10.3, *p* < 0.0001, see Fig. 7E for significance of vapor effect at individual timepoints). There was no significant influence of genotype on ambulation nor on the depressant effect of CIE withdrawal (genotype effect: F_1,12_ = 0.5, *p* = 0.49; genotype x vapor interaction: F_1,12_ = 0.08, *p* = 0.78). To test the function of the intrinsic pacemaker, mice were then switched to constant darkness and chi-square periodogram analysis was used to determine the period length and relative power of the dominant circadian component of ambulation counts (Supplementary Fig. 7). Two-way ANOVA revealed a significant interaction between vapor and genotype on period length (F_1,12_ = 6.5, *p* = 0.025), but none of the pairwise comparisons reached statistical significance. Neither the K361N substitution nor alcohol withdrawal significantly affected the relative power (genotype effect: F_1,12_ = 2.5, *p* = 0.14; vapor effect: F_1,12_ = 2.2, *p* = 0.17; genotype x vapor interaction: F_1,12_ = 0.60, *p* = 0.45).

## Discussion

Altogether, our data demonstrate that BK channel inhibition can modulate alcohol drinking in mice, but the underlying mechanism does not involve the interaction of ethanol with BK α residue K361. This interaction is also not required for alcohol to produce ataxia, sedation, hypothermia, analgesia, and reward, nor for CIE to produce metabolic and locomotor changes during withdrawal.

The major behavioral disturbances elicited by the blockade of BK channels have historically been a hurdle to analyze the behavioral relevance of ethanol’s action at this target. This limitation is illustrated by the results of our pharmacological experiments, whereby the dose-dependent effects of penitrem A on ethanol intake had to be disentangled from tremor induction. The dose of 0.1 mg/kg reduced alcohol drinking both at baseline and following additional weeks of 2BC in air mice, without inducing significant tremor or affecting saccharin drinking. The insensitivity of CIE mice to this dose may reflect adaptive processes producing molecular tolerance to ethanol [12, 46, 53–55], especially in light of the high BECs measured in this cohort, or a possible upregulation of BK channels by CIE, as seen in alcohol-exposed flies [9–11]. In contrast, the dose of 0.05 mg/kg increased alcohol intake. This effect was only detected after additional weeks of 2BC in air mice, suggesting that it requires long-term alcohol drinking and may have been masked by intake escalation in CIE mice (ceiling effect). The dose-dependent pattern of penitrem A’s effects on alcohol drinking may reflect gradually insurmountable blockade (with ethanol acting as a BK channel activator) or priming at 0.05 mg/kg and substitution for ethanol at 0.1 mg/kg (with ethanol acting as a BK channel inhibitor).

Paxilline injected at doses at least ten times lower than doses typically used to induce tremors (6–8 mg/kg, [44, 56]) did not cause overt behavioral abnormalities and did not alter ethanol intake. An even lower dose of paxilline had been previously shown to reverse picrotoxin- and pentylenetetrazole-induced seizures in the absence of tremors [16], which suggests that the doses we used were high enough to significantly reach and block BK channels in the mouse brain, but we cannot rule out that higher doses would have replicated the effects of penitrem A. It is also possible that penitrem A exerted its behavioral effects via a different target that was spared by paxilline. Notably, penitrem A can increase GABA release, reduce GABA uptake, and bind GABA_A_ receptors [57, 58], which are another direct target of ethanol [59, 60] and could conceivably mediate the effect of penitrem A on alcohol drinking. Consistent with this scenario, BK channel activation by BMS-204352, at a dose known to acutely reverse the sensory hypersensitivity and social interaction deficits of *Fmr1* KO mice [17, 18], had no effect on ethanol intake.

The mHb is a known hotspot for the expression of BK channel α, β4, and γ3 subunits in the brain [61–63]. Using oligoprobes specific for BK α alternatively spliced exons STREX and ALCOREX, the mHb was the mouse brain area showing the strongest signal. The presence of β4 and ALCOREX both confer ethanol sensitivity to BK channels [46, 64], making the mHb well-suited for the validation of the K361N substitution. To the best of our knowledge, the effects of paxilline or ethanol on mHb BK channels had never been reported. We found that inhibition of mHb BK channels by paxilline lowered the spike threshold, as previously described in dentate gyrus granule cells [65]. Spike width and afterhyperpolarization, two parameters regulated by BK channels in other neuronal populations (see [66] for review), had inconsistent sensitivity to paxilline in WT mHb neurons. Ethanol’s effects in WT slices were similar to those of paxilline (reduced spike threshold, no effect on other parameters), suggesting that ethanol may inhibit BK channels in the mHb, as previously suggested in the central amygdala [40]. The lack of effect of ethanol on spike threshold in KI slices supports a direct mechanism and confirms the resistance of K361N channels to ethanol in a native setting. Several factors extrinsic to the site of ethanol’s interaction with BK α are known to influence the direction of ethanol’s effect on BK channel activity (potentiation vs. inhibition), such as posttranslational modification of BK α, auxiliary subunits associated with BK α, local calcium and magnesium concentrations, and the lipid environment [64, 67–70]. Accordingly, it is conceivable that both the potentiating and inhibitory effects of ethanol are sensitive to the K361N substitution, albeit this was only demonstrated for potentiation in vitro [15]. We recognize that single-channel recordings would provide a more direct readout of ethanol’s effect on BK channel function in the mHb.

Preventing ethanol from interacting with BK α via the K361N substitution did not significantly alter the acute behavioral responses to alcohol examined here in mice. This finding contrasts with the ability of a genetic manipulation in worms (T381I substitution) to reduce the effects of acute ethanol on egg laying and locomotion [7]. The homologous mammalian residue (T352) is adjacent to the alcohol-sensing site identified by Bukiya et al. [15], but the effect of mutating this residue remains to be tested in mice.

The lack of a consistent effect of K361N on CIE-induced ethanol intake escalation is also at odds with the phenotypes of BK β1 and BK β4 knockout mice we had reported in this paradigm [13]. Based on the known influence of BK β1 and β4 subunits on the potentiation of BK-mediated currents by ethanol [64], our previous results suggested that the action of ethanol at BK channels promotes escalation. We thus expected that the K361N substitution would hinder escalation. The negative outcome reported here suggests that the influence of BK channels on alcohol drinking might be indirect (e.g., recruitment of BK channels downstream of another primary target of ethanol). This scenario would be compatible with intact alcohol drinking in KI mice, given that the K361N substitution does not impair the physiological activity of BK channels [15]. Alternatively, the role of BK channels in the regulation of alcohol drinking may be mediated by BK channel auxiliary subunits and/or by a BK α site(s) different from K361. This scenario is relevant to alcohol-induced constriction of cerebral arteries, which is mediated by the inhibition of myocyte BK channels and requires BK β1 [71] but is unchanged in BK α K361N KI mice (Kuntamallappanavar G, Bukiya AN, and Dopico AM; unpublished results).

We hypothesized that, aside from alcohol drinking escalation, ethanol’s action at BK channels may mediate other physiological consequences of CIE exposure. We detected significant effects of CIE on metabolism, which, to the best of our knowledge, had never been reported in this mouse model. We found that 4 weeks of CIE significantly altered the body composition of mice, reducing fat content and increasing lean content without affecting their total body mass. This observation is consistent with reports of reduced body fat in chronic alcoholics, in the absence of body weight change and in proportion to the level of alcohol consumption [72–75]. The leaner phenotype of CIE-exposed mice was associated with a significant increase in food intake during the first week of withdrawal, which may reflect a homeostatic adaptation to the loss of body fat. Withdrawal from CIE was also associated with a robust increase in RER, reflecting preferential utilization of carbohydrates as a fuel. This pattern may result from deficient lipid storage, as reflected by reduced body fat, and a corresponding inability to sustain normal levels of fatty acid oxidation. In humans, chronic alcohol abuse increases daily caloric intake, yet alcohol represents a substantial fraction of this intake, such that energy intake provided only by food ingestion is typically lower than in healthy counterparts, and normalizes after 14 days of abstinence [72, 73, 76]. Likewise, AUD is associated with lower respiratory quotient, higher lipid oxidation, and reduced carbohydrate oxidation, which all normalize after three months of abstinence [72, 73, 76, 77]. However, to the best of our knowledge, the possibility that a rebound increase in food intake and respiratory quotient may occur during the first week of abstinence has not been explored in humans. Altogether, these observations may be relevant to the state of metabolic ketosis resulting from increased acetate metabolism in AUD and the ability of a ketogenic diet to alleviate somatic withdrawal and craving during the first week of detoxification [78]. In any case, the phenotype of KI mice indicates that the action of ethanol at BK α K361 is not responsible for the metabolic and nutritional changes associated with early withdrawal from chronic alcohol exposure.

One limitation of the pharmacological and genetic manipulations used in the present study is that they were systemic and constitutive, respectively, which may have occluded phenotypic changes if BK channels expressed in different brain regions exert opposing effects on a given alcohol-driven behavior. Further complicating the situation, ethanol has differential effects on BK channel activity in different neuronal populations, owing at least in part to the complement of auxiliary subunits expressed by these cells: activation in neurohypophysial terminals [1, 39], nucleus accumbens somas [2], and external globus pallidus Npas1 neurons [3]; putative inhibition in central amygdala terminals [40] and mHb somas (this study); no effect in supraoptic nucleus cell bodies [39] and nucleus accumbens dendrites [2]. These region- and subcellular compartment-specific subpopulations of BK channels may in turn be differentially sensitive to the effects of pharmacological modulators. Accordingly, the effect of a ligand or mutation in a given brain region or neural circuit may offset its effect elsewhere. Another important consideration is that the present investigation focused on effects of alcohol that we hypothesized to be mediated by neuronal BK channels. However, BK channels are also expressed by other brain cell types, with particularly high levels found in astrocytes and oligodendrocyte precursor cells [66, 79, 80]. Future studies will aim to determine whether alcohol’s effects on the function of glial cells might depend on the interaction of ethanol with BK α K361.

In conclusion, our data show that, in the mouse, ethanol’s interaction with BK α K361 does not mediate the role of BK channels in the modulation of moderate and excessive alcohol drinking. This interaction is also not required for acute responses to various doses of ethanol or for the metabolic consequences of CIE exposure. It remains possible, however, that the action of ethanol at BK α K361 mediates molecular, cellular, or behavioral effects of acute or chronic alcohol that were not examined in the present study.

### Supplementary information

Supplementary Fig.s

## Data Availability

All data supporting the findings of this study are available within the article and its supplementary information files.
